# Restrained expansion of the recall germinal center response as biomarker of protection for influenza vaccination in mice

**DOI:** 10.1371/journal.pone.0225063

**Published:** 2019-11-14

**Authors:** Laurens P. Kil, Joost Vaneman, Joan E. M. van der Lubbe, Dominika Czapska-Casey, Jeroen T. B. M. Tolboom, Ramon Roozendaal, Roland C. Zahn, Harmjan Kuipers, Laura Solforosi

**Affiliations:** Janssen Vaccines & Prevention B.V., Pharmaceutical Companies of Johnson and Johnson, Leiden, The Netherlands; Icahn School of Medicine at Mount Sinai, UNITED STATES

## Abstract

Correlates of protection (CoP) are invaluable for iterative vaccine design studies, especially in pursuit of complex vaccines such as a universal influenza vaccine (UFV) where a single antigen is optimized to elicit broad protection against many viral antigenic variants. Since broadly protective antibodies against influenza virus often exhibit mutational evidence of prolonged diversification, we studied germinal center (GC) kinetics in hemagglutinin (HA) immunized mice. Here we report that as early as 4 days after secondary immunization, the expansion of HA-specific GC B cells inversely correlated to protection against influenza virus challenge, induced by the antigen. In contrast, follicular T helper (TFH) cells did not expand differently after boost vaccination, suggestive of a B-cell intrinsic difference in activation and differentiation inferred by protective antigen properties. Importantly, differences in antigen dose only affected GC B-cell frequencies after primary immunization. The absence of accompanying differences in total anti-HA or epitope-specific antibody levels induced by vaccines of different efficacy suggests that the GC B-cell response upon revaccination represents an early and unique marker of protection that may significantly accelerate the pre-clinical phase of vaccine development.

## Introduction

While antibodies targeting hemagglutinin (HA) protect against influenza virus [1,2], the humoral anti-HA response continuously requires adaptation to newly emerging HA variants. Although seasonal vaccines are currently the most effective measure to prevent influenza, the laborious production of yearly reformulated vaccines prohibits immediate tuning of vaccine HA composition to viral antigenic drift or shift[3], with the associated potential for mismatches between circulating virus strains and the strains included in the vaccine[4]. The resulting suboptimal protection by these vaccines has prompted different approaches to design a universal influenza vaccine (UFV)[5,6,7]. The development of a UFV usually comprises the rational design of numerous vaccine candidates and multiple successive screening rounds of vaccine candidates. Identification of a serological or cellular correlate of protection (CoP) early during the immune response to the HA protein could therefore accelerate UFV development and pandemic preparedness[8].

Although protective efficacy of conventional full-length (FL) HA-based vaccines can be reasonably estimated using hemagglutination inhibition (HAI) titers[9], this assay falls short as CoP for a UFV that does not elicit antibodies interfering with sialic acid binding by the HA head[10,11]. In mouse cohorts vaccinated with either HA stem-derived UFV candidate antigens (“mini-HA”) or FL HA, protective efficacy could not be linked to the mere antibody levels against divergent HA subtypes[12,13]. While follow-up pre-clinical studies indicated that certain stem epitope-specific antibodies may prove a better CoP[12], substantial titers of such antibodies only arise relatively late after multiple vaccinations and therefore are not a suitable CoP to significantly accelerate vaccine candidate screening programs. Instead, as cellular responses precede the rise of protective antibodies, the involved cell subsets and their differentiation stages may represent potential early CoPs.

In recent years, HA-specific antibody analyses have shed light on the cellular characteristics of protective humoral immune responses[14], suggesting a central role for germinal center (GC) responses and memory cell re-engagement[15]. Firstly, broadly neutralizing anti-HA antibodies (bnAbs) are somatically hypermutated, reflecting cognate B and T-cell interactions. Importantly, reverting these bnAbs to their germline configuration disrupts both HA affinity and breadth of binding[16,17]. Secondly, when pre-existing immunity is low, re-engagement and further hypermutation of memory B cells can induce cross-reactive anti-HA responses[18–20]. And thirdly, persistent local GC structures in the lung have been shown to fuel prolonged diversification and enhanced antibody cross-reactivity of memory B cells[21]. Despite this evidence for requirement of (memory) B-cell diversification in GCs for protective humoral responses, some findings challenge the extent of GC expansion required for broadly protective responses. For example, one study has shown that rapamycin treatment of mice during vaccination decreased induction of GCs while improving heterosubtypic protection[22], thereby implicating that protective B-cell responses may also arise in a GC-independent, extrafollicular differentiation route.

Based on these observations we hypothesized that the kinetics of B-cell subsets in GCs or extrafollicular responses could provide early CoPs for the development of a UFV. In this study, we investigated antigen-specific B-cell differentiation kinetics in mice that were immunized with a set of UFV candidates, including stem-based antigens, with known different capacities to induce a protective immune response against lethal influenza virus challenge[13]. We show that restrained GC expansion during the recall response was characteristic for a protective immunization regimen. To our knowledge, this is the first time that the kinetics of germinal center cell subsets can be linked to influenza vaccine efficacy, and this knowledge provides a cellular readout to advance the development of new influenza vaccines and might be applicable to other vaccine development.

## Materials and methods

### Statement of ethics

All mouse experiments were performed at Janssen Vaccines & Prevention B.V., the Netherlands, in accordance with Dutch legislation on animal experiments and approved by the Dutch Dierexperimentencommissie (DEC), an independent ethical institutional review board (Approved DEC protocol numbers CHR35100114, CRH3780115 and CRH24700115). The outcome of the experiments had the potential to accelerate the screening and selection of vaccine condidates and reduce the need to perform animal challenge experimentations. To minimize discomfort, all immunizations were performed under Isofluare anaestesia. Challenged animals were sedated using a ketmine/xylazine solution prior to the intranasal administration of the influenza strain.

Due to the severity of the challenge symptoms in unprotected mice, humane end points were followed to prevent unnessary suffering as much as possible. For the duration of the challenge (21day’s total) mice were monitored daily by experienced operators for bodyweight and clinical scores. Clinical scores were defined as: 0 = no clinical signs, 1 = rough coat, 2 = rough coat, less reactive, passive during handling, 3 = rough coat, rolled up, labored breathing, passive during handling, 4 = rough coat, rolled up, labored breathing, unresponsive. Mice reaching clinical score 4 were euthanized immediately. Score parameters were developed based on observations of previous studies in collaboration with the DEC. Alignment on clinical scores assignment among operators was ensured by in house training and observation by multiple operators. In this study a total of 48 mice were challenged with Influenza virus. In total 26 animals died due to Influenza infection. Six animals were euthanized for reaching clinical score 4, 20 animals were found dead in cage, while the rest of the animals survived the viral challenge.

### HA antigen production and labeling of HA probes

The vaccine HA antigens mini-HA constructs UFV#4157, #4650 and #4900 based on HA A/Brisbane/59/07[13], and H1 FL HA A/Brisbane/59/07 #2316, were produced in-house using HEK293 (Thermo Fisher) cells as described previously [13].

To allow site-directed biotin labeling of the FL HA used as a probe for B cell staining, H1 A/Brisbane/59/2007 was C-terminally modified with a sortase A LPXTG recognition sequence[23]. This modified HA (FL H1#5070) was produced in HEK293F cells cultured in Freestyle^™^ medium (Thermo Fischer, Cat. No. 12338018) by transient transfection using 293fectin^™^ transfection reagent (Invitrogen, Cat. No. 12347019) of the plasmid pcDNA2004 (pcDNA3 vector with an enhanced CMV promoter) encoding FL H1#5070. Culture supernatants were harvested at day 7 by centrifugation followed by filtration over a 0.2 μm bottle top filter (Corning). Proteins were purified in a two-step protocol using an ÄKTA Avant 25 system (GE Healthcare Life Sciences). Sortase-mediated transpeptidation was performed over night (o/n) at 37°C by mixing FL H1#5070 with GGGGGK-Biotin (Pepscan) at a 1:30 molar ratio in reaction buffer (50mM Tris, 150mM NaCl, 10mM CaCl2, 10% Glycerol, pH7.5, 10% Glycerol) supplemented with 0.11 μM Sortase-A enzyme (in house production). Purification of trimeric fractions was performed on SEC-MALS (HPLC: ThermoFisher, detectors: Wyatt) using a Superdex 200 10–300 GL column (GE Healthcare Life Sciences) and purified proteins were stored in 20mM Tris, 150mM, NaCl, pH7.8 buffer at -80°C. Prior to FACS staining, biotinylated probes were separately conjugated for 30 minutes to either APC- or PE-labeled streptavidin at a 3:2 molar ratio (streptavidin to HA), followed by 1:1 mixing of volumes of these streptavidin-labeled fractions for 30 minutes before use in FACS stainings (see “Flow cytometric procedures”).

### Immunization and viral challenge

Six week old female BALB/c mice (specific pathogen-free) were purchased from Charles River laboratories (Sulzfeld, Germany) and allowed to acclimatize for 2 weeks. Depending on experimental design, up to 3 immunizations with FL HA, mini-HA proteins or PBS were administered at 3 week intervals (at days 0, 21 and 42) by intramuscular (i.m.) injection with 100 μl vaccine prepared in PBS (50 μl per hind leg). All immunizations were adjuvanted with Al(OH)_3_, Alum (2% Alhydrogel^®^, Brenntag, Cat. No. 21645-51-2) and performed under full aneastesia with Isoflurane (Abbott). Blood for interim serological analyses was obtained through submandibular bleeding, while blood for serological analyses at experimental endpoints (sacrifice for cellular analyses, humane endpoint during viral challenge, or after 21 days of survival after viral challenge) was obtained through heart puncture under anaesthesia with Isoflurane. Bloods samples were kept in collection tubes for 1 hour at room temperature (RT) to allow clotting to occur, centrifuged (4 minutes @4000RPM followed by 1 minute at 14000RPM), and subsequently serum was isolated. When animals were sacrificed for cellular analyses, draining (iliac) lymph nodes were isolated for cellular phenotyping.

For lethal viral challenge experiments, one day prior to challenge (day 69) a positive control group for survival (n = 8) received intravenously 300 μg of broadly neutralizing antibody CR6261 diluted in PBS. Four weeks after the final immunization (day 70), challenge cohorts (n = 10) were anaesthetized by intraperitoneal (i.p.) administration of 100 mg/kg ketamine in combination with 20 mg/kg xylazine. Mice were challenged with 25xLD_50_ (8.310^4^ 50% Tissue Culture Infective Dose (TCID_50_)) of H1N1 A/Netherlands/602/2009 via the intranasal route (a total of 50 μl; 25 μl per nostril). Bodyweight and clinical scores were monitored daily for up to 21 days post challenge or until the defined humane endpoint was reached to limit animal discomfort. Humane endpoint was defined based on clinical score as is established practice.

### Flow cytometric procedures

Single cell suspensions of lymphoid organs were prepared by straining over a 100 μm filter (BD Falcon) in PBS (Gibco, Life Technologies, Cat. No. 10010–015). Cells were stained in 96-well U-bottom plates (BD Falcon) at 1*10^^6^ cells per well with a mix of titrated fluorochrome conjugated monoclonal antibodies specific for surface markers (see S1 Table) in FACS buffer (PBS, 0.5% BSA, 2 mM EDTA) at 50 μl/well for 45 minutes at RT. Cells were washed three times with 180 μl of PBS and stained for 10 minutes on ice with viability dye diluted in 50 μl PBS/well. After washing three times with 180 μl of PBS, cells were fixated for 60 minutes at 4°C in 100 μl/well of Foxp3 Transcription Factor Fixation buffer (eBioscience, Kit Cat. No. 00-5523-00). Cells were washed with 180 μl/well of Foxp3 Transcription Factor Permeabilization buffer (eBioscience, Kit Cat. No. 00-5523-00) before addition of antibodies against intracellular antigens (see S1 Table) or/and titrated PE/APC conjugated HA probes (see “HA antigen production and of HA probes”) in Foxp3 Transcription Factor Permeabilization buffer. After 30 minutes on ice the cells were washed two times in Foxp3 Transcription Factor Permeabilization buffer and resuspended in 150 μl of FACS buffer. Compensation controls, for hardware compensation, were prepared with cells using fluorochromes listed in S1 Table. Stained samples were acquired without setting an event limit by an LSR Fortessa (BD Biosciences) with Diva software version 8.0.1 and analyzed using FlowJo, versions 7.9 and 9.5.2 (FlowJo, LLC). A minimum of 200 CD19+ or CD4+ events was set as acceptance critirea to consider sample data to be valid.

### Full length HA ELISA

Serum antibodies against the full-length HA protein of H1 A/Brisbane/59/07 and of H1 A/California/07/09 were measured in enzyme-linked immunosorbent assay (ELISA). Maxisorp 96- well plates (Nunc^™^, Thermo Scientific, Cat. No. 439454) were coated o/n with 0.05 μg/well recombinant FL HA (Protein Sciences, Cat. No. 3006H1_A_BRISBANE_59_2007, 3006H1_CALIFORNIA/07/09) in PBS (Gibco, Life Technologies, Cat. No. 10010–015). Using a programmed ELx405 automated plate washer (BioTek) plates were washed 3 times with 300 μl of PBS supplemented with 0.05% Tween-20 (Calbiochem, Merck Millipore, Cat. No. 655204). Wells were blocked for 1 hour at RT with block buffer (PBS,2% skimmed milk; Difco, BD Cat. No. 232100). Following the plate washing step, 10 2-fold serial dilutions of serum samples were prepared in duplicates in block buffer starting with 1:50 dilution. Serum antibodies were allowed to bind to the target protein for 1 hour at RT. Subsequently wells were washed and incubated with a 1:2000 dilution of Goat-anti-Mouse IgG-HRP (KPL, Cat. No. 474–1802) in block buffer for 1 hour at RT. After washing of the plates OPD substrate (Thermo Scientific, Tablets: Cat. No. 34006 Buffer Cat. No. 34062) was added for 10 minutes after which the reaction was stopped by adding 1M H_2_SO_4_. The optical density (OD) was measured at 492 nm by a Powerwave HT plate reader (BioTek), and standard curves were fitted using a four-parameter logistic curve. Conversion of raw OD measurements to ELISA units per ml (EU/ml) and assessments of lower limit of detection (LOD) values were essentially performed as published previously[13]. Briefly, the resulting OD of each sample dilution was quantified against the standard mCR9114 (a chimeric monoclonal antibody consisting of the variable domains of human CR9114 with a mouse IgG2a Fc constant domain, produced in-house). The final concentration per sample (in log_10_ EU/ml) was calculated by a weighted average, using the squared slope of the standard curve at the location of each quantification as weight. All samples below LOD were set at the LOD, defined as the lowest sample dilution multiplied by the lowest standard concentration, with an OD response above the lower asymptote of the standard curve and background.

### CR9114 competition ELISA

To quantify CR9114 epitope-competing antibodies 96-well Maxisorp plates (Nunc^™^, Thermo Scientific) were coated o/n at 4 °C with purified polyclonal rabbit anti His-Tag IgG (GenScript USA Inc. Cat. No. A00174–200). Plates were washed using an ELx405 automated plate washer (BioTek) programmed for 3 washes with 300 μl of PBS supplemented with 0.05% Tween-20 (Calbiochem, Merck Millipore, Cat. No. 655204) and subsequently blocked with 2% BSA in PBS for 1 hour at RT. After washing, the plates were incubated for 2 hours with His-tagged FL HA of A/Brisbane/59/2007 (in-house production), washed again, and serum was added in duplicate followed by a 2-fold serial dilution in blockbuffer. After the first hour of incubation at RT, the competing biotinylated human IgG1 CR9114 was added (0.02μg/ml). The plates were incubated for 1 additional hour at RT and washed again before adding streptavidin-HRP for 1 hour. The plates were washed, developed using OPD substrate (Thermo Scientific, Tablets: Cat. No. 34006 Buffer Cat. No. 34062) and stopped after 10 minutes with 1M H_2_SO_4_. OD was measured at 492nm by a Powerwave HT plate reader (BioTek) and fitted using a 4-parameter logistic curve. The CR9114 competition of each sample was quantified as the slope of the linear regression of OD value on the log10 dilution for the duplicate series.

### Statistical analysis

Statisitcal differences in responses elicited by the selected vaccine candidates were evaluated in B- and T-cell subset frequencies as well as in HA binding antibodies and CR9114-competing antibodies. For flowcytometry the recorded cell frequencies per million live cells were log-transformed and plotted per treatment group, whereas the HA-binding ELISA data was plotted as ELISA units per mL and competition ELISA data as Slope OD. A one-way ANOVA corrected for multiple comparisons using Tukey’s statistical hypothesis testing was applied to compare between group means.

Statistical analyses were performed using GraphPad Prism version 7 (GraphPad Software, La Jolla, CA, USA, www.graphpad.com).

## Results

### Flow cytometric detection and characterization of HA-specific B cells

To examine the murine B-cell response to HA vaccination regimens on a cellular level, we designed an antibody panel for flow cytometric phenotyping of multiple mature B-cell subsets. To distinguish between mature B-cell subsets reflecting differences in functionality and maturity, we included a B-cell lineage marker (CD19), an exclusion marker (CD4), markers for GC differentiation (GL7 and CD95/FAS), a marker for plasmablast and plasma cell differentiation (CD138/syndecan-1), and membrane markers upregulated by subsets of memory B cells (CD80 and PD-L2)[24,25]. To enable reliable detection of low frequency HA-specific B cells in rare B-cell subsets (e.g. plasma cells and subsets of memory B cells) we employed a previously described double antigen staining approach that minimizes false-positive designation of antigen-specific B cells by the exclusion of fluorochrome-binding cells[26,27]. As labeling antigen for antigen-specific B cells, an HA-probe (H1#5070) derived from A/Brisbane/59/07 (H1#2316) was designed to allow site-directed biotin conjugation, reminiscent of approaches published earlier[23,28].

**Fig 1:**
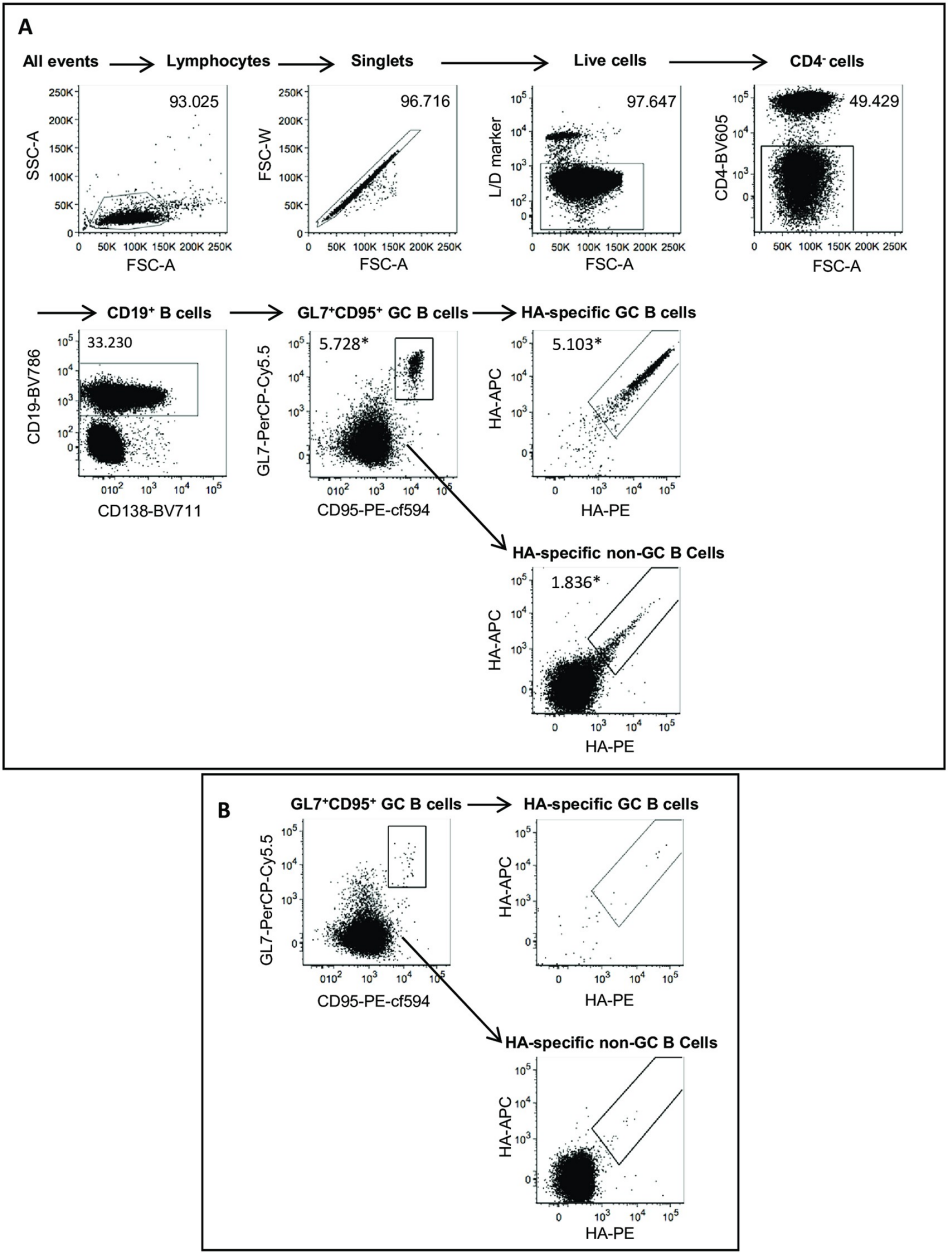
The applied flowcytometric B-cell phenotyping panel is able to identify HA specificity within B-cell subsets. Mouse iliac lymph node cells were obtained 19 days post-prime immunization with alum adjuvanted FL H1#2316 (A) or PBS (B). Cells were subsequently stained for CD4, CD19, CD138, GL7, CD95, and a biotinylated HA probe (FL H1#5070) conjugated to APC (HA-APC) and PE (HA-PE) fluorochromes was used to assess HA binding among B cell subsets. Gate frequencies are indicated as frequency of parent or, if followed by a “*”, as frequency of CD19+ B-cells. Arrows drawn from gates to plots show the applied sequential gating steps to identify the cell populations shown in plot titles in (B) all gating steps prior to the identification of B cells are not shown. Data are representative for n = 8 immunized mice.

To verify whether antigen-specific B cells can be detected at increased frequencies in HA-immunized animals, we immunized 8 weeks-old influenza-naive mice with either FL H1#2316 or PBS adjuvanted with alum and subsequently analyzed the B-cell response in draining (iliac) lymph nodes using the described flow cytometric phenotyping panel. In draining lymph nodes from HA-immunized mice we could detect double-positive B cells binding both APC- and PE-labeled FL H1#5070 among GC (CD19^+^GL7^+^CD95^+^) and non-GC B-cell populations (CD19^+^GL7^-^CD95^-^) see Fig 1A. Affirming that these cells are indeed HA-specific B cells induced by immunization, these double positive B cells among these cell populations was minimal or undetected in PBS-immunized mice (Fig 1B). Taken together, these analyses show that our flow cytometric approach allows the phenotypical characterization of mature B-cell subsets while concomitantly determining the HA-specificity of these cells.

### Germinal center kinetics after primary immunization do not predict vaccine-mediated protection

**Fig 2:**
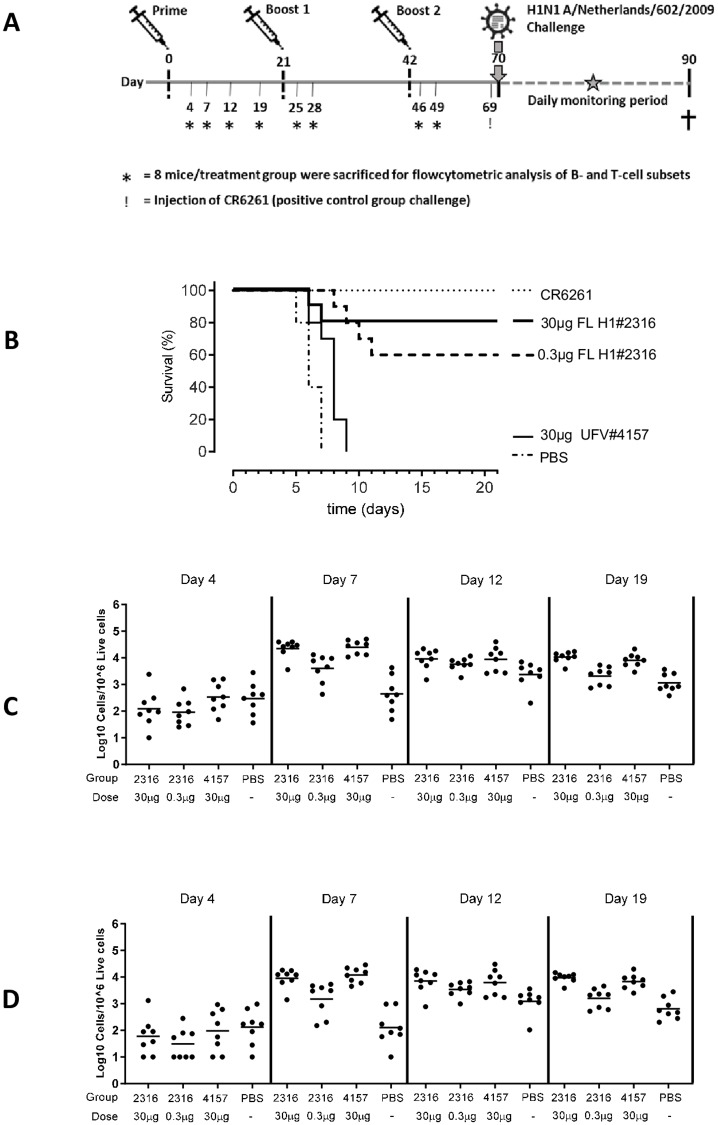
Germinal center B cell kinetics post-prime immunization do not correlate to vaccine protective efficacy. Following the immunization schedule (A) a large cohort of mice were immunized three times at three week intervals (day 0, 21 and 42) with 30 μg alum-adjuvanted FL H1#2316, 0.3 μg alum-adjuvanted FL H1#2316, 30 μg alum-adjuvanted UFV#4157, or PBS alum-adjuvanted. At the indicated days (4, 7, 12 and 19 post-prime immunization), 8 mice per treatment group were sacrificed and iliac lymph nodes used for FACS analysis. The remaining mice (n = 10 per immunized cohorts, n = 8 for positive control CR6261 cohort) went trough a lethal influenza virus challenge on day 70 (25xLD50 of H1N1 A/Netherlands/602/2009) and were monitored for 21 days. A positive control group (n = 8) for influenza virus protection was intravenously injected with 300 μg of bnAb CR6261 at day 69. The challenge outcome is displayed as Kaplan-Meier survival (B). Relative counts of GC B cell subsets in iliac lymph nodes (quantified using the flow cytometric analysis outlined in Fig 1A) were measured at the indicated time-points post-prime immunization in mice (n = 8 per time-point per cohort) vaccinated with 30 μg alum-adjuvanted FL H1#2316, 0.3 μg alum-adjuvanted FL H1#2316, 30 μg alum-adjuvanted UFV#4157, or PBS alum-adjuvanted. Graphs in (C) show the total counts of GL7+CD95+ B cells while in (D) GL7+CD95+ B cells binding to both FL H1#5070-PE and FL H1#5070-APC conjugates are included. Each symbol represents one animal while group means are indicated by a horizontal bar.

Next, we investigated whether the kinetics of mature B-cell subsets after immunization can predict the protective efficacy of an immunization regimen. For this, we compared B-cell subset frequencies between cohorts of mice subjected to immunization regimens inducing different levels of protection against lethal H1N1 influenza virus challenge. Naïve 8-weeks old mice were immunized three times, at days 0, 21 and 42 with different immunogens. One cohort of animals was vaccinated with a high dose (30 μg) of a trimeric full-length H1 HA antigen (FL H1#2316) that provides heterologous protection against H1N1 A/Netherlands/602/2009, while a second cohort received a minimally protective vaccine containing a high dose (30 μg) of an HA stem-based monomeric antigen (UFV#4157) derived from FL H1#2316 [13]. To control for differences in immune responses related to the dose rather than intrinsically protective properties of the used immunization antigen, a lower dose (0.3 μg) of FL H1#2316, which was expected to be partially protective, was used for immunizations of a third cohort, while as a negative control for survival a fourth cohort was immunized with PBS only. All immunizations, including mock-immunizations with PBS, were adjuvanted with alum. To analyze cellular responses at early and late timepoints of GC reactions after prime or boost immunizations, 8 animals per cohort were sacrificed at 8 different timepoint (days 4, 7, 12, 19, 25, 28, 46 and 49) (Fig 2A). To assess the protective efficacy of the administered vaccine regimens, 10 animals per immunized cohort and 8 animals of the positive control group that passively immunized with the broadly neutralizing antibody CR6261, were challenged intranasally with a lethal dose of 25xLD_50_ H1N1 A/Netherlands/602/2009, heterologous to the parental H1 virus strain, to determine the protective efficacy of each vaccination regimen. After influenza virus challenge, we observed that high (30 μg) and low (0.3 μg) doses of FL H1#2316 conferred survival rates of 80% and 60% respectively, while a high dose (30 μg) of UFV#4157 failed to provide any protection (Fig 2B). All the animals in the positive control group survived the influenza virus challenge, while all the animals that received PBS immunization succumbed to the infection (Fig 2B).

In search for differences in GC responses between FL H1#2316 and UFV#4157 cohorts, from day 7 onwards after prime immunization we found clear GC induction by FL H1#2316 and UFV#4157 immunizations which persisted at days 12 and 19 (Fig 2C). While in the three immunization cohorts high frequencies of HA-specific cells were detected among GC B cells (Fig 2D), both total and HA-specific GC B-cell numbers appeared to be lower in the 0.3 μg FL H1#2316 cohort. These results indicate that the magnitude of the primary GC B-cell response is largely governed by the antigen dose, rather than other immunizing vaccine properties including its induced protective efficacy.

### Instrinsic antigen properties shape the GC recall response

**Fig 3:**
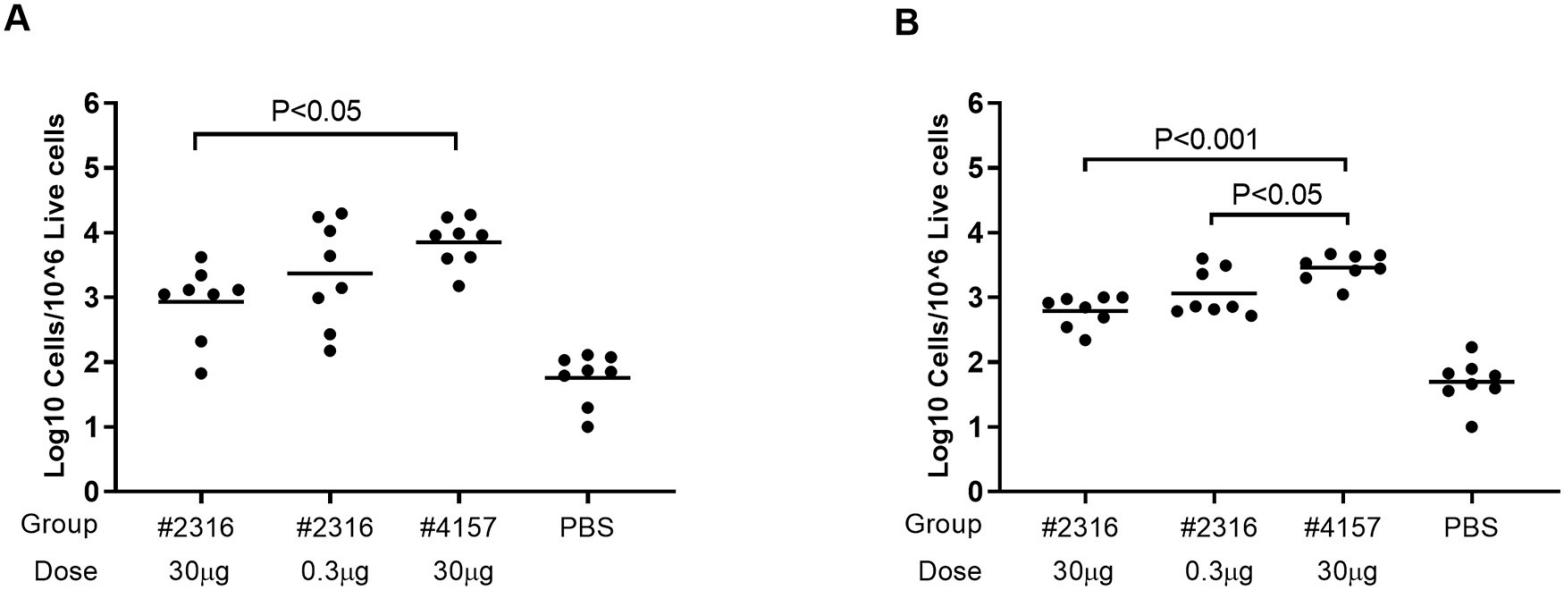
Increased GC B cell expansion after boost-immunization with a non-protective UFV#4157 immunization. Frequencies of cells binding to both FL H1#5070-PE and FL H1#5070-APC conjugates (HA+) among GC (GL7+CD95+) B cells (A) and non-GC (GL7-CD95-) B cells (B) in iliac lymph nodes were measured 4 days post-boost (day 25) in mice (n = 8 per time-point per cohort) vaccinated with 30 μg alum-adjuvanted FL H1#2316, 0.3 μg alum-adjuvanted FL H1#2316, 30 μg alum-adjuvanted UFV#4157 or alum-adjuvanted PBS. Each symbol represents one animal while group means are indicated by a horizontal bar. Statistical comparisons are made by comparing group means of the immunized groups in a one-way ANOVA, corrected for multiple comparisons using Tukey’s statistical hypothesis testing.

As GC kinetics after primary immunization could not discriminate between a protective and an inert vaccine regimen, we continued to study the GC response in the same cohorts after boost immunizations. As early as 4 days after the first boost immunization (day 25), we noticed that the low dose FL H1#2316 immunization cohort displayed comparable, if not slightly higher, numbers of (HA-specific) GC B cells in the draining lymph nodes compared to the high dose FL H1#2316 immunizations (Fig 3A). In contrast, the cohort immunized with a high dose of the non-protective UFV#4157 antigen showed a significantly larger expansion with an 8-fold difference in HA-specific GC B cells (Fig 3A). The same trend was observed for HA-specific non-GC B cells, with a 4-fold difference between the cohort immunized with FL H1# 2316 and the one immunized with UFV#4157 (Fig 3B). Although HA-specific non-GC B cells do not exclusively comprise bona fide memory B cells (as no single murine marker has been identified to indisputably identify memory cells), the vast majority of HA-binding non-GC B cells must represent memory B antigen-experienced cells as the frequencies of these non-GC B cells were well below 0.01% in the PBS-immuzed cohort (Fig 3B). Further analyses of PD-L2 and CD80 expression as well as Ig isotype expression among HA-binding non-GC B cells did not reveal consistent expression differences at post-boost time-points between immunized cohorts (S1 Fig).

To determine whether the enhanced GC recall response was restricted to the first boost vaccination response only, we also quantified HA-specific GC B cells in the draining lymph nodes 4 days after the second boost vaccination (day 46). Again, the same enhanced expansion of GC B cells was observed in the UFV#4157 immunized cohort (S2A Fig). A similar trend was observed for HA-specific non-GC B cells (S2B Fig). Notably, both at days 28 (1 week post first boost) and 49 (1 week post second boost), HA-specific GC B-cell frequencies were again comparably high between cohorts immunized with high doses of FL H1#2316 or UFV#4157 (S3 Fig), suggesting an accelerated rather than a generally increased expansion of GCs after boost immunizations. After the second boost immunization, GC B-cell frequencies were once again not significantly different between the low versus high dose FL H1#2316 immunized cohorts (S3 Fig).

Given this marked difference in B-cell expansion 4 days post boost, we also examined whether GC-associated T-cell subsets followed a similar expansion pattern. To study follicular T helper (TFH) cells and regulatory (FoxP3^+^) TF (TFR) cells in draining iliac lymph nodes, a flow cytometric gating strategy was employed to respectively discern TFH and TFR populations as CD4^+^CXCR5^+^Foxp3^-^PD-1^high^CCR7^low^ICOS^high^Bcl6^high^ and CD4^+^CXCR5^+^PD1^+^Foxp3^+^ cells (S4 Fig). However, neither at day 25 nor 46 a significant difference in cell frequencies of TFH or TFR populations could be observed between the immunized groups (S5 Fig).

Finally, to examine whether observed differences in post-boost GC B cell expansion were possibly mirrored by changes in anti-HA antibody responses, we measured antibody levels against the homologous HA immunization antigen (A/Brisbane/59/07) as well as against heterologous HA from A/California/07/09 (which shares 99.4% sequence homology with the HA of the used challenge strain A/Netherlands/602/09). While anti-A/Brisbane/59/07 HA antibody titers were higher in both high-dose and low-dose FL H1#2316 immunized cohorts compared to the UFV#4157 immunizedcohort (S6A Fig), this potential biomarker for heterologous protection has been disqualified previously as protective HA stem-derived antigens (UFV#4900 [13]) have been reported to induce lower anti-A/Brisbane/59/07 HA antibody levels than FL HA, due to the absence of induction of antibodies targeting immunodominant HA head epitopes [12]. Also, antibody levels against A/California/07/09 did not mirror differences in protection by FL H1#2316 and UFV#4157 immunizations as the development of these antibody titers was largely comparable between all immunized cohorts (S6B Fig), with only a modest delay in induction of these antibodies in low-dose FL H1#2316 immunized mice.

In summary, the expansion of GC B cells, rather than the expansion of GC-related T-cell subsets shortly after boost immunizations or the levels of anti-HA antibodies, appears to differ with the type of antigen used. As FL HA antigen FL H1#2316 and HA-stem derived UFV#4157 greatly differ in structural properties, it remains unclear whether differences in the GC recall response can be attributed to differences in protective properties or merely to structural differences.

### GC recall response inversely correlates with the protective nature of immunization antigens

In contrast to the headless HA-stem-derived antigen UFV#4157, the FL HA antigen #2316 induces high antibody levels to immunodominant HA head epitopes which may explain why a strong and successful primary GC response in the (high dose) FL H1#2316 cohort is followed by a focused and relatively smaller GC expansion shortly after boost immunization. In addition, these head-binding antibodies induced in FL H1#2316 immunized mice may also provide some protection against a heterologous challenge as some conserved epitopes can also be found on the HA head region[29].

Therefore, to determine whether the magnitude of the GC recall response indeed reflects protective efficacy conferred by the immunization antigen and not structural differences per se, we compared GC responses induced by structurally closely related antigens with known differences in the level of protective efficacy. For this, we immunized cohorts of mice using the same prime-boost scheme with three mini-HA antigens: monomeric UFV#4157, mostly dimeric UFV#4650 and trimeric UFV#4900 at 30 μg/dose. These mini-HA’s are all derived from the same A/Brisbane/59/07 HA stem, but they confer different levels of heterologous and heterosubtypic protection against lethal influenza challenge in mice[13], ranging from minimal to partial to broad protection against heterologous group 1 strains for UFV#4157, UFV#4650 and UFV#4900 respectively. To enable differentiation between GC effects induced by the presence versus the absence of HA head epitopes and GC effects truly reflecting protective versus non-protective immune responses, we included a cohort immunized with FL H1#2316 at equimolar levels (60 μg/dose). A mock (PBS) immunization cohort was again included as unprotected reference group.

**Fig 4:**
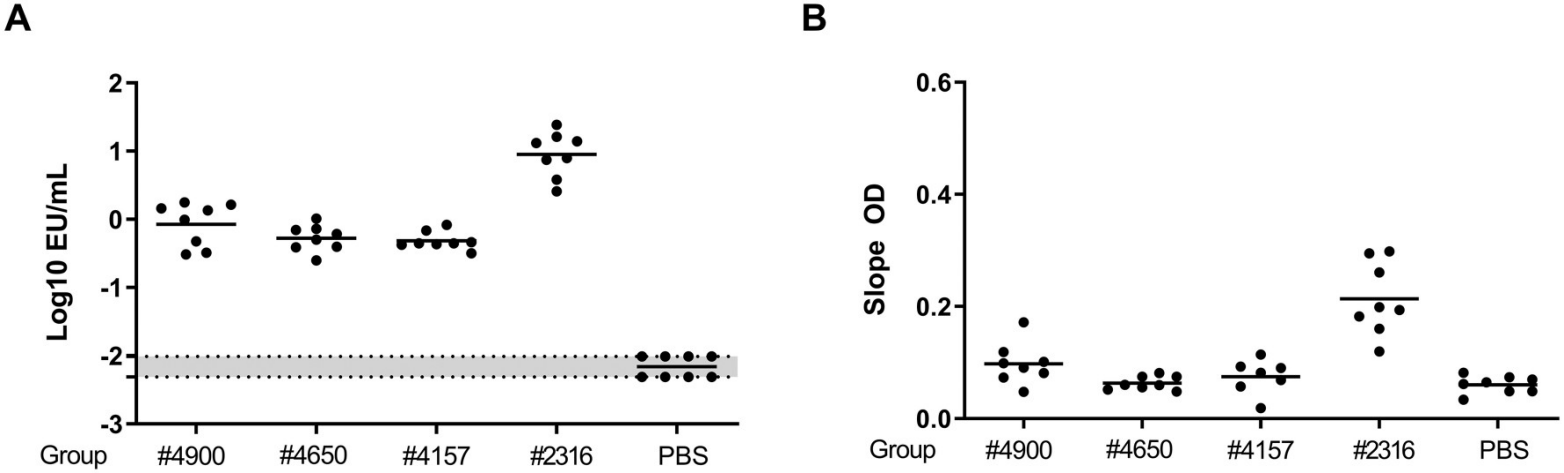
Early post-boost immunization antibodies do not correlate with protection conferred by mini-HA immunizations. (A) ELISA titers against full-length HA derived from A/Brisbane/59/07 (same antigen as FL H1#2316) were determined in serum obtained at day 25 (4 days after the first boost immunization) from mice (n = 8 per group) immunized with high doses (30 μg or 60 μg) of the indicated antigens or PBS, all adjuvanted with alum. (B) Inhibition of CR9114 binding to A/Brisbane/59/07 determined by ELISA after pre-incubation of A/Brisbane/59/07 with day 25 post-boost serum antibodies from the same mice. Every dot represents data from a single animal, horizontal bars specify group means. In panel A, the grey area between dotted lines represents the highest and lowest LOD of the assay, which is calculated per each plate. Statistical comparisons are made by comparing group means of the miniHA vaccinated animals in a one-way ANOVA, corrected for multiple comparisons using Tukey’s statistical hypothesis testing.

**Fig 5:**
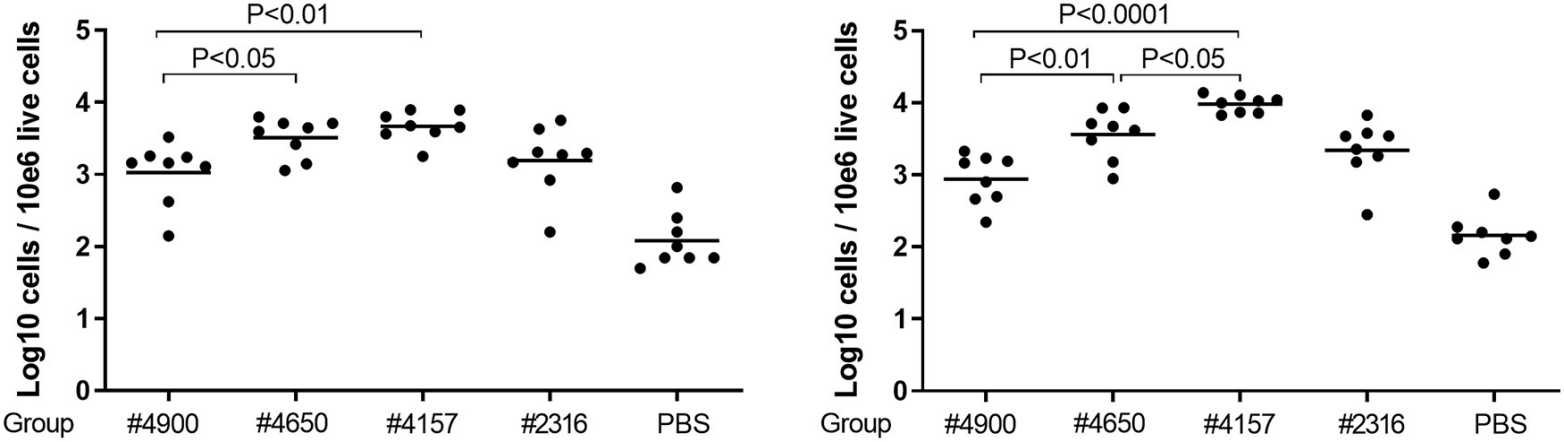
Protective immunization regimens induce lower HA-specific B cells responses post-boost immunizations. Frequencies of HA-binding cells among GC B cell (A) and non-GC B cell (B) populations in iliac lymph nodes as measured at day 25 (4 days post-boost immunization) in mice (n = 8 per time-point per cohort) vaccinated with the alum-adjuvanted Mini-HA UFV#4900, UFV#4650, UFV#4157 antigens (30 μg), FL HA FL H1#2316, at an equimolar dose of 60 μg, or PBS. Each symbol represents one animal while group means are indicated by a horizontal bar. Statistical comparisons are made by comparing group means of the miniHA vaccinated animals in a one-way ANOVA, corrected for multiple comparisons using Tukey’s statistical hypothesis testing.

Since we aim to identify a marker of protection early after immunization (in contrast to late HA stem-binding antibodies[12], and since only day 25 and day 46 revealed significant differences in HA-reactive B-cell frequencies in the previous experiments, we confined our analyses to day 25 only. As expected, based on the immunodominance of the HA head, 4 days post-boost immunization full-length FL H1#2316 immunization induced higher serum levels of total anti-HA antibodies than any HA stem-derived antigen (Fig 4A). Additionally, while antibodies competing for the CR9114 HA-stem epitope were detectable at low levels in FL H1#2316-immunized mice, significant induction of these antibodies at day 25 was still absent in the mini-HA-immunized cohorts (Fig 4B). In contrast, at day 25 marked differences in HA-specific GC and non-GC B cells numbers were observed (Fig 5A and 5B). Immunizations with the protective UFV#4900 antigen induced comparable frequencies of these cells 4 days post-boost compared to FL H1#2316, while frequencies were significantly increased for UFV#4650 and UFV#4157 immunized mice, resulting respectively in a 3- and 4- fold increase compared to UFV4900 in HA specific GC B-cells and a 4- and 10- fold increase for HA specific non-GC B cells.

In summary, these experiments show that the recall GC response after secondary immunization can serve as an inverse biomarker to predict the greatly different protective efficacies of structurally related antigens.

## Discussion

The quest for CoPs in vaccinology is a long-standing one. Rather than one single biomarker to predict the efficacy of a vaccine as early as possible after immunization, it seems more likely that different CoPs could emerge depending on the use of different pathogens, antigens, doses, adjuvants and many other factors. Rather than analyzing whether we could find one universal CoP predicting efficacy of all possible vaccines, we instead focused specifically on identifying an early biomarker to significantly accelerate the laborious process of iterative *in vivo* screenings of antigens such as the mini-HAs developed for a UFV[13]. These mini-HAs were designed to most stably and truthfully mimic the HA stem epitope targeted by bnAbs such as CR9114[30]. The discovery of CR9114-competing antibodies as pre-clinical CoP in heterologous lethal influenza virus challenge[12] demonstrates the successful rationale chosen for mini-HA design. Despite a strong correlation to survival, this CoP does not infallibly predict survival in each individual animal. Moreover, this CoP was only assessed four weeks after a third immunization, thereby barely shortening tedious *in vivo* screenings of vaccine candidates and thus prompting our search for an earlier CoP.

In this study, we show that as early as four days after the second vaccination with an HA antigen, an exaggerated expansion of antigen-specific GC B cells corresponds to a low or absent protective efficacy. We found that differently protective immunizations with structurally closely related antigens (mini-HAs) show different GC B-cell expansions while similarly protective immunizations with structurally different antigens (FL HA versus mini-HA) show similar post-boost GC B-cell expansions. Together, these finding directly link an exaggerated post-boost expansion of GC B cells to unprotective properties of the antigen, and not to other structural characteristitcs of the antigen. This exaggerated GC recall response was not mirrored by an equally enhanced expansion of TFH cells or related T-cell subsets, suggesting a selective difference in activation, differentiation or selection of antigen-specific B cells.

While the mechanism driving enhanced post-boost GC B-cell expansion by suboptimal vaccine antigens or dose has not been unraveled in this study, many of the potential mechanisms point towards an early immunological imprint after prime immunization affecting the expansion of GC B cells post-boost immunization. The more restrained GC B cell expansion induced by more protective antigens might be caused by higher post-prime levels of (high-affinity) antibodies. Higher antibody titers could for example shield immunodominant epitopes during the recall response from antigen-specific B cells or modulate B cell activation through engagement of their Fc receptors by antigen complexes[31]. As differences in pre-boost HA-specific antibody levels did not correlate with protection and the GC recall response, and since pre-boost CR9114-competing antibodies were virtually absent in all cohorts (S7 Fig), mere levels of epitope-specific antibody levels prior to boost vaccination are unlikely to decisively affect the recall GC B-cell response. In contrast, other antibody Fc properties (such as isotype and glycosylation patterns) affecting antibody affinity and breadth of protection of the recall response[32,33] may have shaped the GC recall response in our mouse cohorts. Therefore, profiling Fc characteristics after prime immunization with mini-HA antigens may shed light on selection pressures exerted by prime-induced anti-HA antibodies.

Apart from feedback by pre-existing antibodies, recall responses may alternatively be shaped by the GC seeding capacity of the established memory B-cell compartment, as differences in antibody affinity among these HA-binding cells may have critically shifted GC versus extrafollicular differentiation decisions upon antigen re-engagement[34]. If affinity maturation is indeed suboptimal for unprotective vaccine antigens due to intrinsic antigen properties, this hampered affinity maturation is likely not confined to the primary response and should also affect the recall response. In this light, the exaggerated expansion of GCs in the recall response may resemble enlarged GCs observed due to frustrated affinity maturation as seen for example in activation-induced cytidine deaminase (AID) deficiency[35].

Which antigenic characteristic pivotal for protection could frustrate affinity maturation to alter the affinity of recall GC seeding memory B cells and enhance the expansion of low-affinity GC B cells entering recall GCs? Studies performed on HA and HA-derived stem antigens indicate that the conformational stability of epitopes could be a decisive factor, as rigid epitopes provide more stable templates for evolving GC B cells to iteratively test newly acquired affinities. Indeed, the improved protective efficacy of the mini-HA vaccine candidates used in this study has previously been correlated to increased stability of antigen conformations based on deuterium exchange rates[13]. In addition, the increased avidity provided by multimeric mini-HAs such as UFV#4900 compared to monomeric mini-HAs such as UFV#4157 may aid affinity-based selection of GC B cells. Finally, increased antigen stability and multimeric conformation may positively affect antigen half-life *in vivo*, perhaps contributing to a longer evolution of GC B-cell clones.

Taken together, our findings reveal recall GC B-cell responses to be an early cellular marker associated with protection for HA vaccination studies. Further elucidating the mechanisms shaping the recall GC response may provide a linked CoP that can predict protective efficacy even earlier and in addition possibly serologically, which would allow true CoP analyses on an individual rather than a cohort level. Extensive characterization of the Fc make-up of the anti-HA antibody pool after prime immunization to profile the antibody repertoire of the established memory B cell compartment and their affinities, would further help unravel the mechanisms shaping the recall GC response. In addition, serological markers such as CXCL13[36] or circulating TFHs[37,38] which may truthfully mirror the extent of the GC B cell recall response in draining lymph nodes, could as well support a better understanding of the B cell maturation dynamic. Combined with the use of closely related immunogens, such studies could also reveal whether findings from the current study may be extended beyond the field of HA vaccinations.

## Supporting information

S1 Table Antibodies and fluorescent reagents used in flowcytometry staining. (DOCX)

S1 Fig PD-L2 and CD80 expression among HA-binding non-GC B cells did not reveal consistent expression differences at post-boost time-point (day 25) between immunized cohorts. (TIF)

S2 Fig Increased GC B cell expansion after the second boost-immunization with a non-protective UFV#4157 immunization. Frequencies of cells binding to both FL H1#5070-PE and FL H1#5070-APC conjugates (HA+) among GC (GL7+CD95+) B cells (A) and non-GC (GL7-CD95-) B cells (B) in iliac lymph nodes were measured 4 days after the second boost (day 46) in mice (n = 8 per time-point per cohort) vaccinated with 30 μg alum-adjuvanted FL H1#2316, 0.3 μg alum-adjuvanted FL H1#2316, 30 μg alum-adjuvanted UFV#4157 or alum-adjuvanted PBS. Each symbol represents one animal while group means are indicated by a horizontal bar. Statistical comparisons are made by comparing group means of the immunized groups in an one-way ANOVA, corrected for multiple comparisons using Tukey’s statistical hypothesis testing.
(TIF)

S3 Fig HA-specific GC B-cell frequencies measured on day 28 (1 week post first boost) and 49 (1 week post second boost), show no differences between immunized cohorts. (TIF)

S4 Fig Gating strategy applied in flow cytometric analysis of TFH and TFR cell frequencies. Mouse iliac lymph node cells were obtained 19 days post-prime immunization with alum adjuvanted FL H1#2316 were stained for CD4, CD19, CXCR5, PD1, CCR7, Bcl6, ICOS and Foxp3 to discern follicular T helper (TFH) cells and regulatory TF (TFR) cells. Cell frequencies in the gate are indicated as frequency of parent or, if followed by a “*”, as frequency of CD4+ B-cells Arrows from gates to plots indicate the sequential gating steps applied to quantify these populations. Plot titles indicate the populations shown in plots. Data are representative for n = 8 immunized mice.
(TIF)

S5 Fig Comparable post-boost GC T cell subset frequencies between vaccination regimens with different protective efficacy. At day 25 (4 days post first boost) (A) and at day 46 (4 days post second boost) (B) post immunizations, frequencies of true TFH cells (CD4+CXCR5+Foxp3-CCR7-PD1+Bcl6+ICOS+) and TFR cells (CD4+CXCR5+PD1+Foxp3+) were measured in iliac lymph nodes from mice (n = 8 per time-point per cohort) vaccinated with 30 μg alum-adjuvanted FL H1#2316 (circles), 0.3 μg alum-adjuvanted FL H1#2316 (squares), 30 μg alum-adjuvanted UFV#4157 (upward triangles) or alum-adjuvanted PBS (downward triangles). Each symbol represents one animal while group means are indicated by a horizontal bar.
(TIF)

S6 Fig Kinetics of antibody responses do not mirror differences in protection by FL H1#2316 and UFV#4157 immunizations. ELISA titers against (A) full-length HA derived from A/Brisbane/59/07 (same antigen as FL H1#2316) and against (B) full-length HA derived from A/California/07/09 (which shares 99.4% sequence homology with the HA of the used challenge strain A/Netherlands/602/09), were determined in serum obtained at day 4, 7, 12, 19, 25, 28, 46, 49 and 68 post immunization from mice (n = 8 or 10 per group) immunized with high or low doses (30 μg or 0.3 μg) of the FL H1#2316, with the UFV#4157 or PBS, all adjuvanted with alum. Every dot represents data from a single animal, horizontal bars specify group means. The grey area between dotted lines represents the highest and lowest LOD of the assay, which is calculated per each plate.
(TIF)

S7 Fig CR9114 competing antibodies are absent in serum taken 19 days post prime in all immunized cohorts. (TIF)
