# Supplementary material for: Restrained expansion of the recall germinal center response as biomarker of protection for influenza vaccination in mice
Source: PLoS One. 2019 Nov 14;14(11):e0225063. doi: 10.1371/journal.pone.0225063 (PMC6855462; doi:10.1371/journal.pone.0225063)
Supplement: S1 Table — (DOCX) [file pone.0225063.s001.docx]

**S1 Table.**

**This is the S1 Table Title. Antibodies and fluorescent reagents used in flowcytometry staining.**

| **Fluorescent reagents used in staining panel for B cell analysis** | | | | | |
| --- | --- | --- | --- | --- | --- |
| **Reagent** | **Clone** | **Manufacturer** | **Cat#** | **Dilution** | **Staining** |
| CD4-BV605 | RM4-5 | BD Biosciences | 563151 | 1:400 | Extracellular |
| CD19-BV786 | 1D3 | BD Biosciences | 563333 | 1:800 | Extracellular |
| CD138-BV711 | 281-2 | BD Biosciences | 563193 | 1:1600 | Extracellular |
| CD80 BV650 | 16-10A1 | BD Biosciences | 563687 | 1:200 | Extracellular |
| CD95-PEcf594 | Jo2 | BD Biosciences | 562499 | 1:100 | Extracellular |
| PD-L2-BV421 | TY25 | BD Biosciences | 564245 | 1:100 | Extracellular |
| GL7-PerCP-cy5.5 | GL7 | Biolegend | 144609 | 1:100 | Extracellular |
| IgM- APC-ef780 | II/41 | eBioscience | 47-5790-82 | 1:200 | Intracellular |
| IgG1-FITC | A85-1 | BD biosciences | 553443 | 1:1600 | Intracellular |
| IgG2a-FITC | R19-15 | BD biosciences | 553390 | 1:800 | Intracellular |
| IgG2b-FITC | R12-3 | BD biosciences | 553395 | 1:1600 | Intracellular |
| IgG3-FITC | R40-82 | BD biosciences | 553403 | 1:400 | Intracellular |
| ZombieAqua | N/A | Biolegend | 423101 | 1:400 | Extracellular |
| Streptavidin-PE | N/A | eBioscience | 12-4317-87 | NA* | Conjugation to HA probe |
| Streptavidin-APC | N/A | eBioscience | 17-4317-82 | NA* | Conjugation to HA probe |
| CD16/CD32 (Fc-block) | 2.4G2 | BD Biosciences | 553142 | 1:100 | Extracellular |
| * PE and APC labelled Streptavidin was conjugated to biotinylated HA probes prior to intracellular staining. Used dilution for conjugated probes (after mixing PE and APC labelled probes): 1:50 | | | | | |
|  | | | | | |
| **Fluorescent reagents used as compensation controls for B cell analysis.** | | | | | |
| **Reagent** | **Clone** | **Manufacturer** | **Cat#** | **Dilution** | **Staining** |
| CD38-FITC | 90 | eBioscience | 11-0381-81 | 1:100 | Extracellular |
| GL7-PerCP-cy5.5 | GL7 | Biolegend | 144609 | 1:100 | Extracellular |
| CD25-APC | PC61.5 | eBioscience | 17-0251-82 | 1:80 | Extracellular |
| IgM- APC-ef780 | II/41 | eBioscience | 47-5790-82 | 1:200 | Extracellular |
| PD1/CD279- BV421 | J43 | BD Biosciences | 562584 | 1:100 | Extracellular |
| ZombieAqua | N/A | Biolegend | 423101 | 1:400 | Extracellular |
| CD4-BV605 | RM4-5 | BD Biosciences | 563151 | 1:400 | Extracellular |
| CD80 BV650 | 16-10A1 | BD Biosciences | 563687 | 1:200 | Extracellular |
| CD138-BV711 | 281-2 | BD Biosciences | 563193 | 1:1600 | Extracellular |
| CD19-BV786 | 1D3 | BD Biosciences | 563333 | 1:800 | Extracellular |
| CXCR5- PE | 2G8 | BD Biosciences | 561988 | 1:20 | Extracellular |
| CD95-PEcf594 | Jo2 | BD Biosciences | 562499 | 1: 100 | Extracellular |
|  | | | | | |
| **Fluorescent reagents used in staining panel for T cell analysis** | | | | | |
| **Reagent** | **Clone** | **Manufacturer** | **Cat#** | **Dilution** | **Staining** |
| CD19- BV605 | 1D3 | BD Biosciences | 563148 | 1:100 | Extracellular |
| CD4- BV786 | RM4-5 | BD Biosciences | 563727 | 1:800 | Extracellular |
| CXCR5- PE | 2G8 | BD Biosciences | 561988 | 1:20 | Extracellular |
| ICOS- FITC | 7E.17G9 | eBioscience | 11-9942-80 | 1:400 | Extracellular |
| CD25- PerCP-Cy5.5 | PC61.5 | eBioscience | 45-0251-82 | 1:400 | Extracellular |
| CCR7- PE-CF594 | 563596 | BD Biosciences | 563596 | 1:20 | Extracellular |
| Foxp3- PE-Cy7 | FJK-16s | eBioscience | 25-5773-80 | 1:160 | Intracellular |
| Bcl6-Alexa Fluor® 647 | K112-91 | BD Biosciences | 561525 | 1:80 | Intracellular |
| PD1/CD279- BV421 | J43 | BD Biosciences | 562584 | 1:100 | Extracellular |
| ZombieNIR | N/A | Biolegend | 423105 | 1:800 | Extracellular |
| CD16/CD32 (Fc-block) | 2.4G2 | BD Biosciences | 553142 | 1:100 | Extracellular |
|  | | | | | |
| **Fluorescent reagents used as compensation controls for T cell analysis.** | | | | | |
| **Reagent** | **Clone** | **Manufacturer** | **Cat#** | **Dilution** | **Staining** |
| CD19- BV605 | 1D3 | BD Biosciences | 563148 | 1:100 | Extracellular |
| CD4- BV786 | RM4-5 | BD Biosciences | 563727 | 1:800 | Extracellular |
| CXCR5- PE | 2G8 | BD Biosciences | 561988 | 1:20 | Extracellular |
| CD38-FITC | 90 | eBioscience | 11-0381-81 | 1:100 | Extracellular |
| CD25- PerCP-Cy5.5 | PC61.5 | eBioscience | 45-0251-82 | 1:400 | Extracellular |
| CCR7- PE-CF594 | 563596 | BD Biosciences | 563596 | 1:20 | Extracellular |
| Foxp3- PE-Cy7 | FJK-16s | eBioscience | 25-5773-80 | 1:160 | Intracellular |
| Bcl6-Alexa Fluor® 647 | K112-91 | BD Biosciences | 561525 | 1:80 | Intracellular |
| PD1/CD279- BV421 | J43 | BD Biosciences | 562584 | 1:100 | Extracellular |
| ZombieNIR | N/A | Biolegend | 423105 | 1:800 | Extracellular |
| CD16/CD32 (Fc-block) | 2.4G2 | BD Biosciences | 553142 | 1:100 | Extracellular |
