# Supplementary figures and images for: Restrained expansion of the recall germinal center response as biomarker of protection for influenza vaccination in mice
Source: PLoS One. 2019 Nov 14;14(11):e0225063. doi: 10.1371/journal.pone.0225063 (PMC6855462; doi:10.1371/journal.pone.0225063)

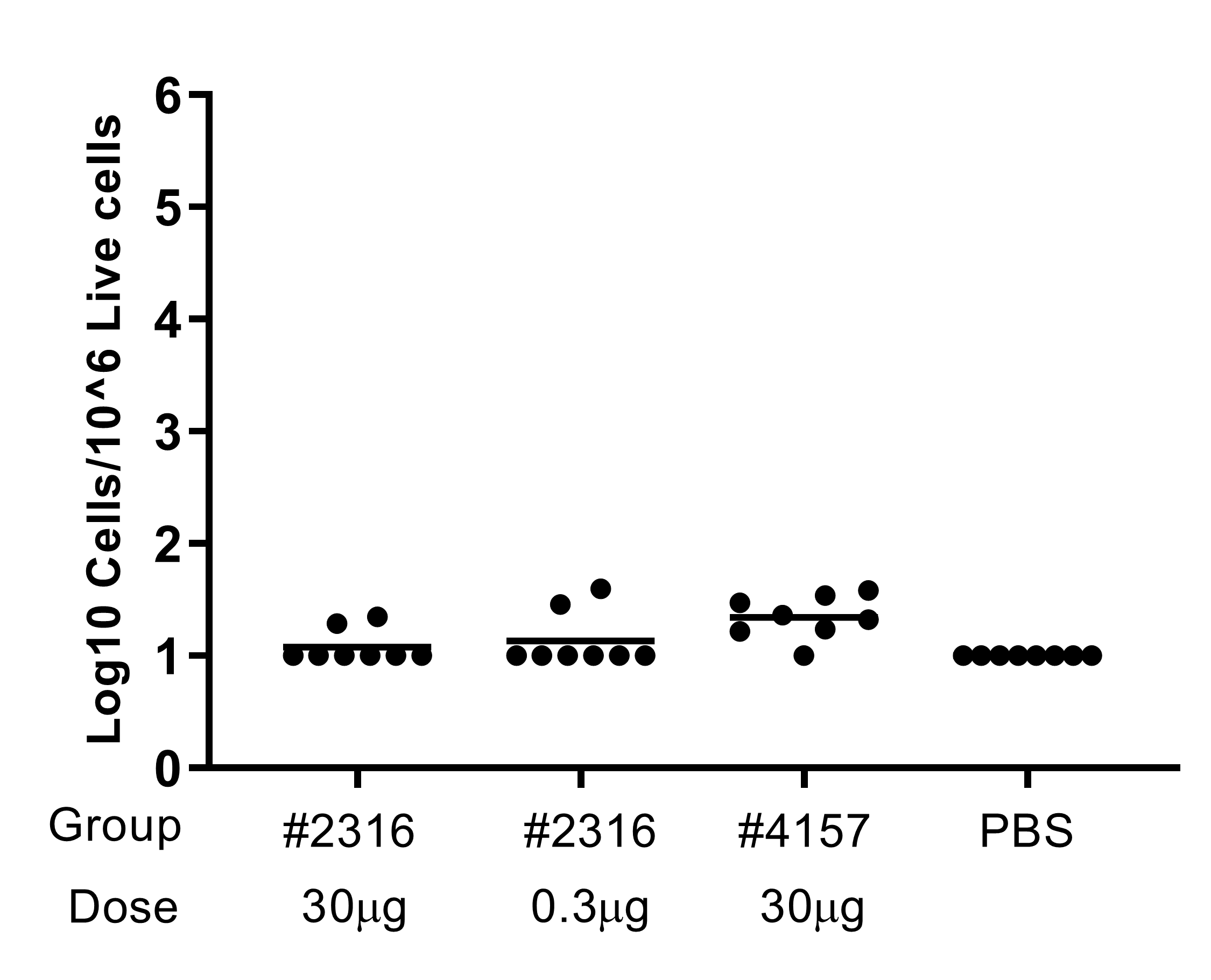

Supplement: S1 Fig — (TIF) [file pone.0225063.s002.tif]

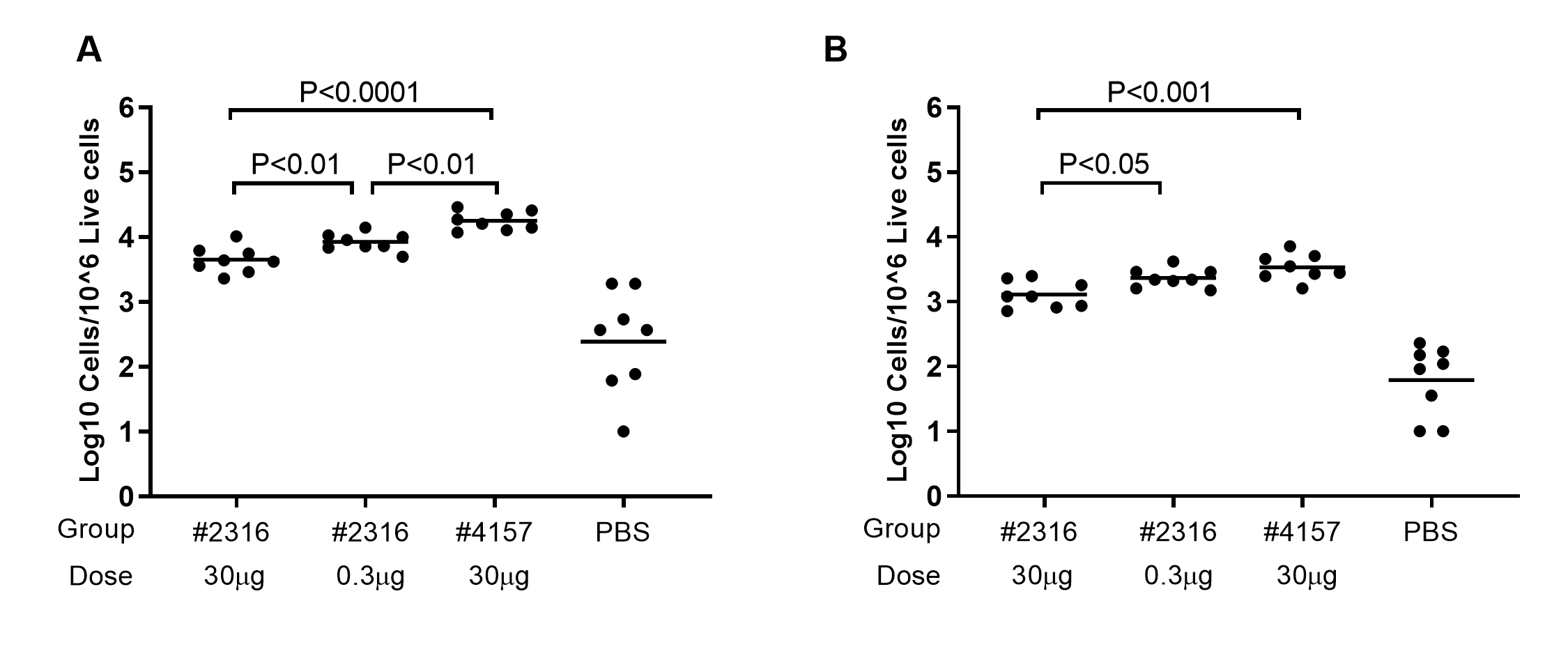

Supplement: S2 Fig — Frequencies of cells binding to both FL H1#5070-PE and FL H1#5070-APC conjugates (HA+) among GC (GL7+CD95+) B cells (A) and non-GC (GL7-CD95-) B cells (B) in iliac lymph nodes were measured 4 days after the second boost (day 46) in mice (n = 8 per time-point per cohort) vaccinated with 30 μg alum-adjuvanted FL H1#2316, 0.3 μg alum-adjuvanted FL H1#2316, 30 μg alum-adjuvanted UFV#4157 or alum-adjuvanted PBS. Each symbol represents one animal while group means are indicated by a horizontal bar. Statistical comparisons are made by comparing group means of the immunized groups in an one-way ANOVA, corrected for multiple comparisons using Tukey’s statistical hypothesis testing. (TIF) [file pone.0225063.s003.tif]

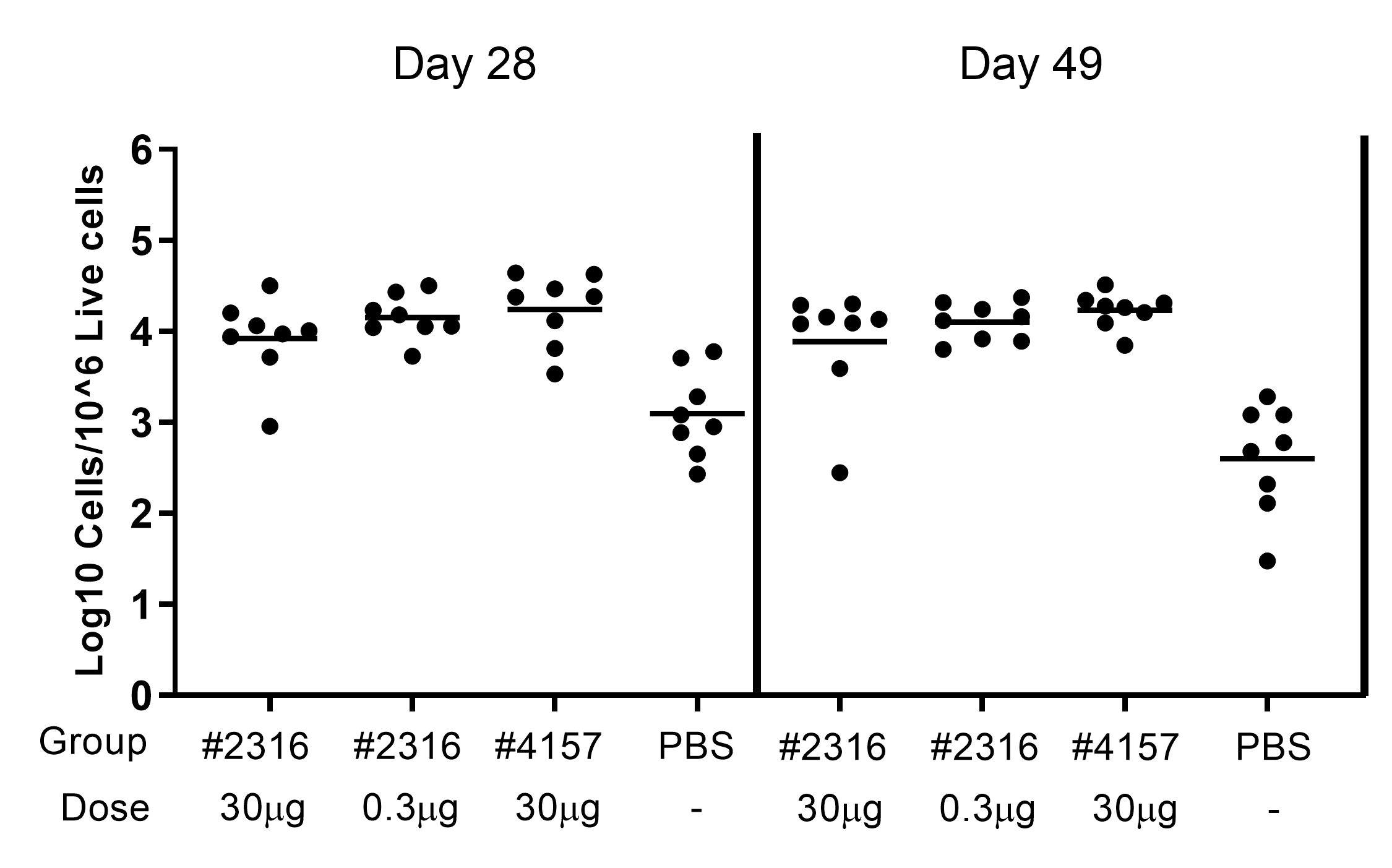

Supplement: S3 Fig — (TIF) [file pone.0225063.s004.tif]

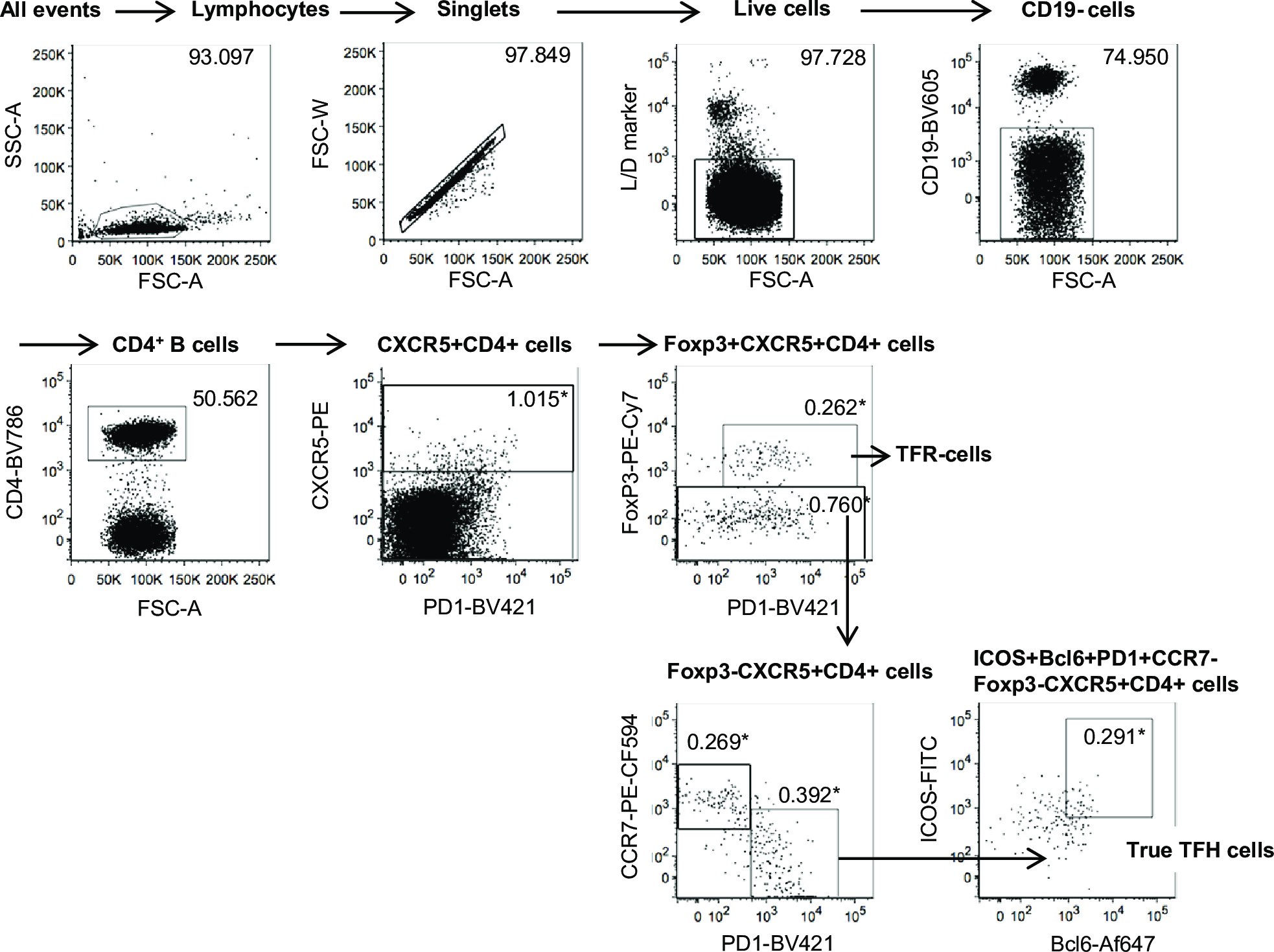

Supplement: S4 Fig — Mouse iliac lymph node cells were obtained 19 days post-prime immunization with alum adjuvanted FL H1#2316 were stained for CD4, CD19, CXCR5, PD1, CCR7, Bcl6, ICOS and Foxp3 to discern follicular T helper (TFH) cells and regulatory TF (TFR) cells. Cell frequencies in the gate are indicated as frequency of parent or, if followed by a “*”, as frequency of CD4+ B-cells Arrows from gates to plots indicate the sequential gating steps applied to quantify these populations. Plot titles indicate the populations shown in plots. Data are representative for n = 8 immunized mice. (TIF) [file pone.0225063.s005.tif]

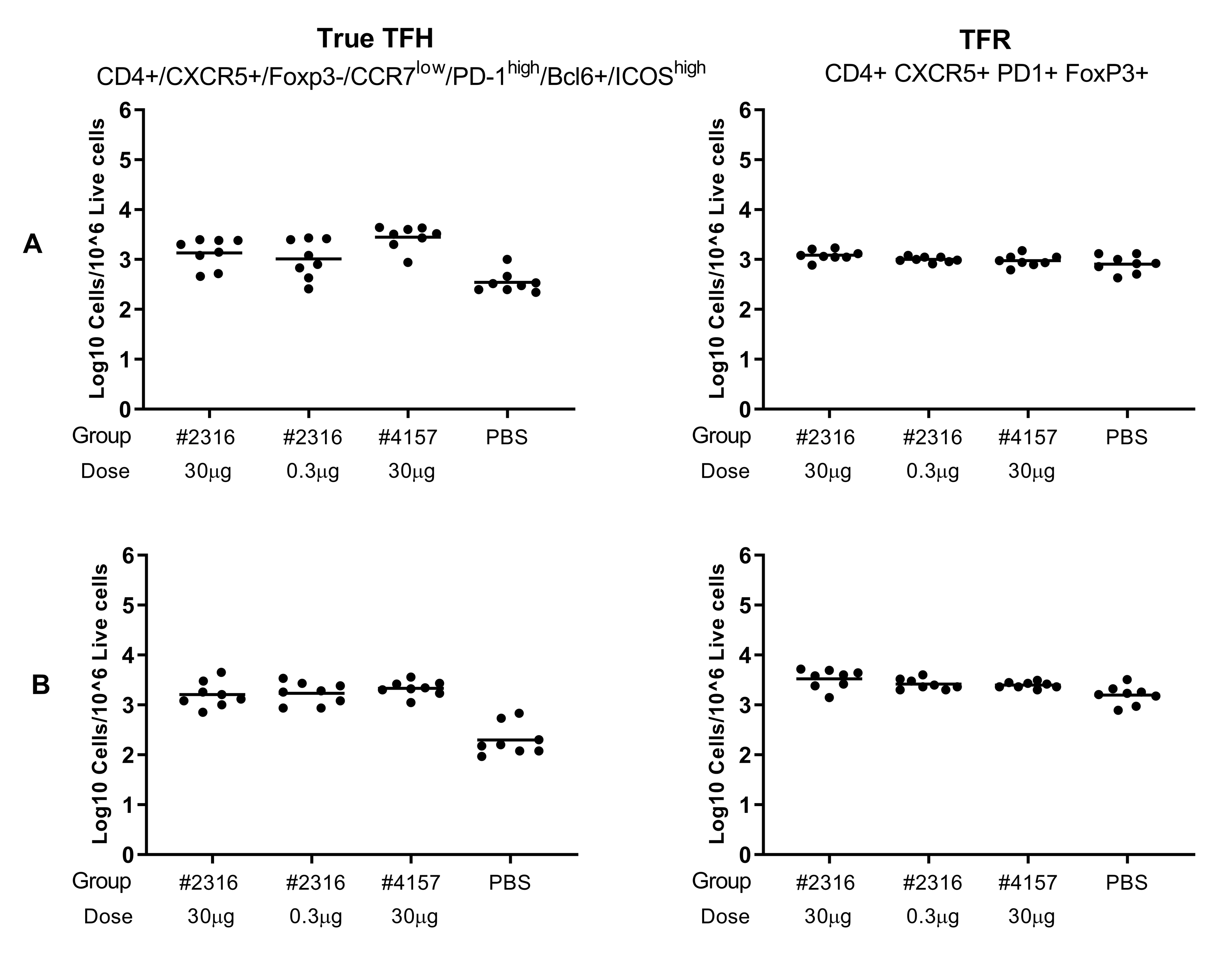

Supplement: S5 Fig — At day 25 (4 days post first boost) (A) and at day 46 (4 days post second boost) (B) post immunizations, frequencies of true TFH cells (CD4+CXCR5+Foxp3-CCR7-PD1+Bcl6+ICOS+) and TFR cells (CD4+CXCR5+PD1+Foxp3+) were measured in iliac lymph nodes from mice (n = 8 per time-point per cohort) vaccinated with 30 μg alum-adjuvanted FL H1#2316 (circles), 0.3 μg alum-adjuvanted FL H1#2316 (squares), 30 μg alum-adjuvanted UFV#4157 (upward triangles) or alum-adjuvanted PBS (downward triangles). Each symbol represents one animal while group means are indicated by a horizontal bar. (TIF) [file pone.0225063.s006.tif]

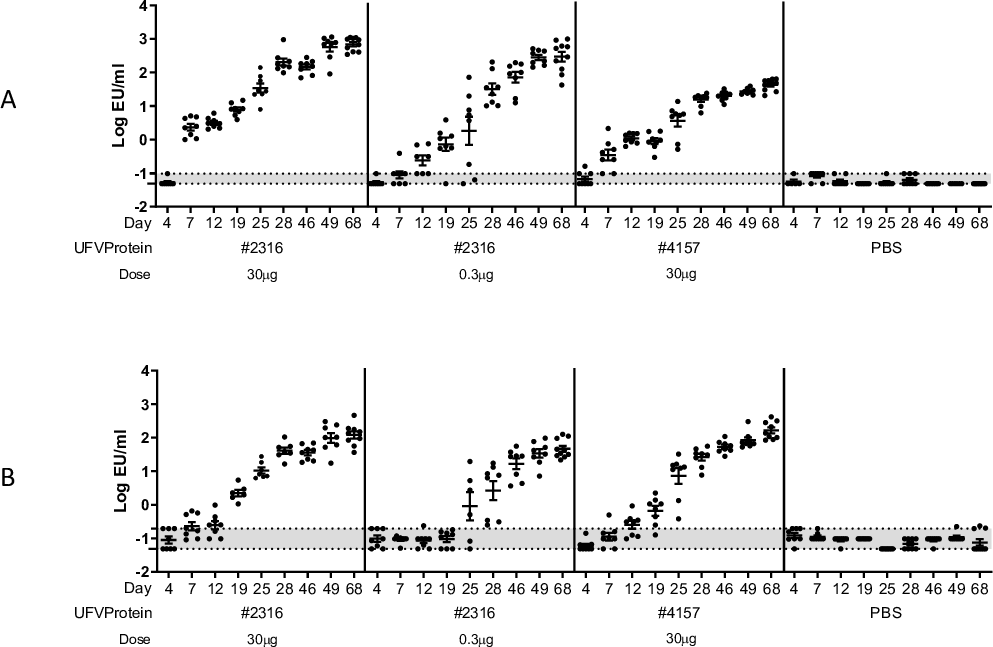

Supplement: S6 Fig — ELISA titers against (A) full-length HA derived from A/Brisbane/59/07 (same antigen as FL H1#2316) and against (B) full-length HA derived from A/California/07/09 (which shares 99.4% sequence homology with the HA of the used challenge strain A/Netherlands/602/09), were determined in serum obtained at day 4, 7, 12, 19, 25, 28, 46, 49 and 68 post immunization from mice (n = 8 or 10 per group) immunized with high or low doses (30 μg or 0.3 μg) of the FL H1#2316, with the UFV#4157 or PBS, all adjuvanted with alum. Every dot represents data from a single animal, horizontal bars specify group means. The grey area between dotted lines represents the highest and lowest LOD of the assay, which is calculated per each plate. (TIF) [file pone.0225063.s007.tif]

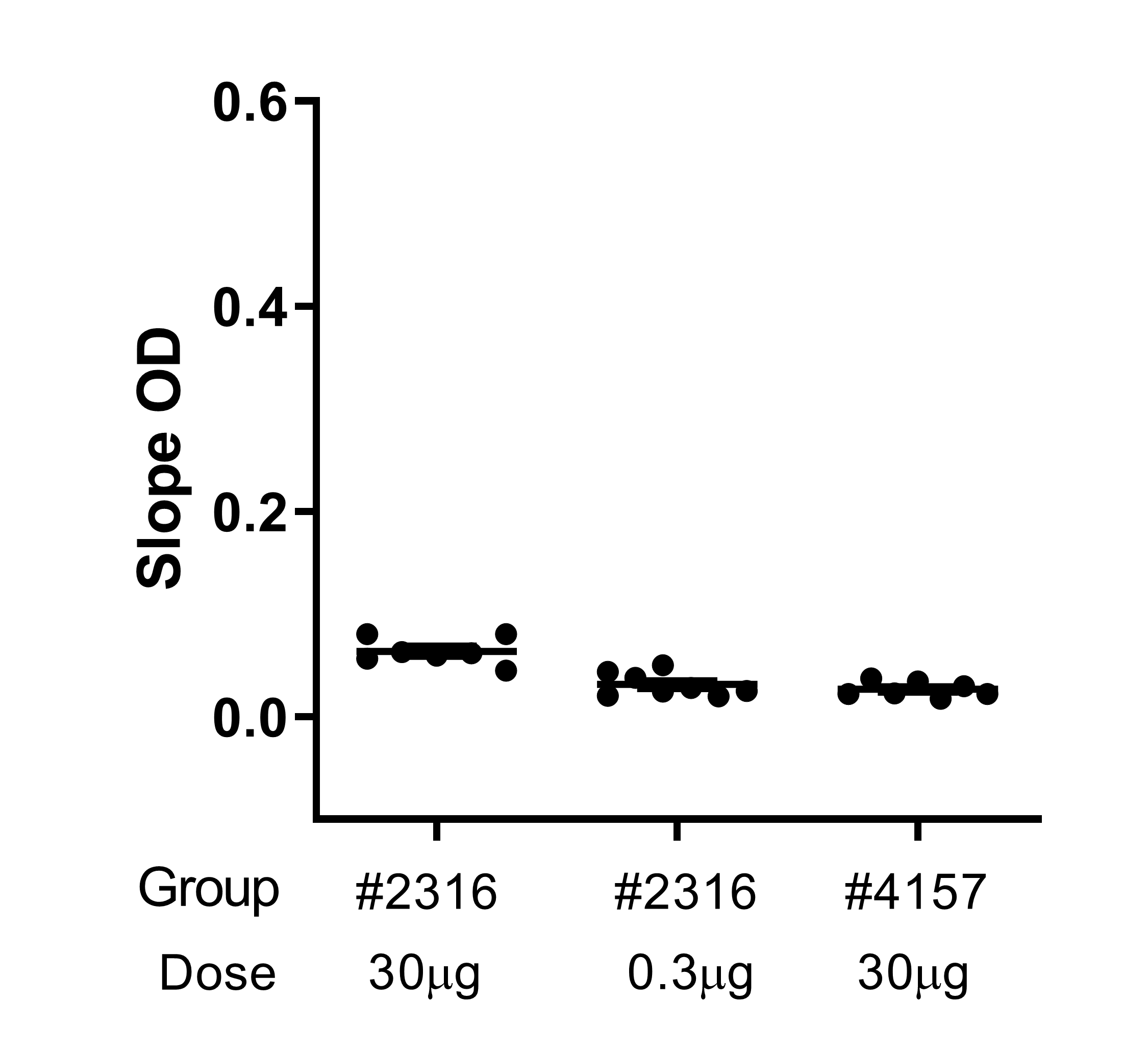

Supplement: S7 Fig — (TIF) [file pone.0225063.s008.tif]
